# Location of AI risk markers and associated mammographic features in screening mammograms obtained years before screen-detected breast cancer

**DOI:** 10.1007/s00330-026-12689-z

**Published:** 2026-06-18

**Authors:** Marit A. Martiniussen, Marie B. Bergan, Merete U. Kristiansen, Solveig Roth Hoff, Henrik Wethe Koch, Fredrik A. Dahl, Solveig Hofvind

**Affiliations:** 1 Department of Radiology and Nuclear Medicine, Østfold Hospital Trust, Kalnes, Norway; 2 Institute of Clinical Medicine, University of Oslo, Oslo, Norway; 3 Department of Breast Cancer Screening, Cancer Registry, Norwegian Institute of Public Health, Oslo, Norway; 4 Department of Radiology, Division of Diagnostics, Møre og Romsdal Hospital Trust, Ålesund, Norway; 5 Department of Health Sciences in Ålesund, Faculty of Medicine and Health Scienses, Norwegian University of Science and Technology, Trondheim, Norway; 6 Department of Radiology, Stavanger University Hospital, Stavanger, Norway; 7 Norwegian Computing Center, Oslo, Norway; 8 Department of Health and Care Sciences, UiT, The Artic University of Norway, Tromsø, Norway

**Keywords:** Breast neoplasms, Mammography, Mass screening, Artificial intelligence

## Abstract

**Objectives:**

To examine screening mammograms assigned high-risk scores by two artificial intelligence (AI) models, 2 and 4 years prior to screen-detected cancers.

**Materials and methods:**

This retrospective study was based on data from 130,031 screening examinations performed in BreastScreen Norway (2008–2018), processed by two AI models: a commercial model (A) and an in-house model (B). The study sample included women with screen-detected cancer, and two prior consecutive screening examinations assigned the highest 10% of AI risk scores. Two radiologists conducted an informed review of three consecutive screening mammograms per woman, 4 and 2 years prior to, and at diagnosis. Descriptive statistics assessed the location of AI markings, mammographic features, histopathological tumor characteristics, and visibility of malignancy preceding diagnosis.

**Results:**

Model A and B assigned high-risk scores at both prior mammograms for 43 and 47 cases, respectively, with 29 cases selected by both, yielding 61 sets of three consecutive examinations. AI markings corresponded to the cancer location in at least one view in 61% and 57% of cases 4 years prior to diagnosis for models A and B, respectively, while radiologists classified 89% as true negative or minimal sign non-specific. At diagnosis, spiculated mass (28%) and density with calcifications (20%) were the most frequent mammographic features but were absent 2 and 4 years prior.

**Conclusion:**

The AI models identified breast cancer in a substantial part of screening mammograms 2 and 4 years preceding diagnosis, but only a few demonstrated suspicious findings according to radiologists.

**Key Points:**

***Question*** *Understanding the correspondence between AI markings, cancer location, and mammographic features is important to evaluate AI models’ potential to enhance early breast cancer detection.*

***Findings*** *AI markings corresponded to the cancer location 4 and 2 years prior to diagnosis, with evolving mammographic features, yet radiologists classified most examinations as negative.*

***Clinical relevance*** *The discrepancy between AI-identified breast cancers years before diagnosis and radiologists classifying the same cases as negative highlights both AI’s potential and the challenges of how best to implement it to enhance early detection.*

**Graphical Abstract:**

**Figure:**
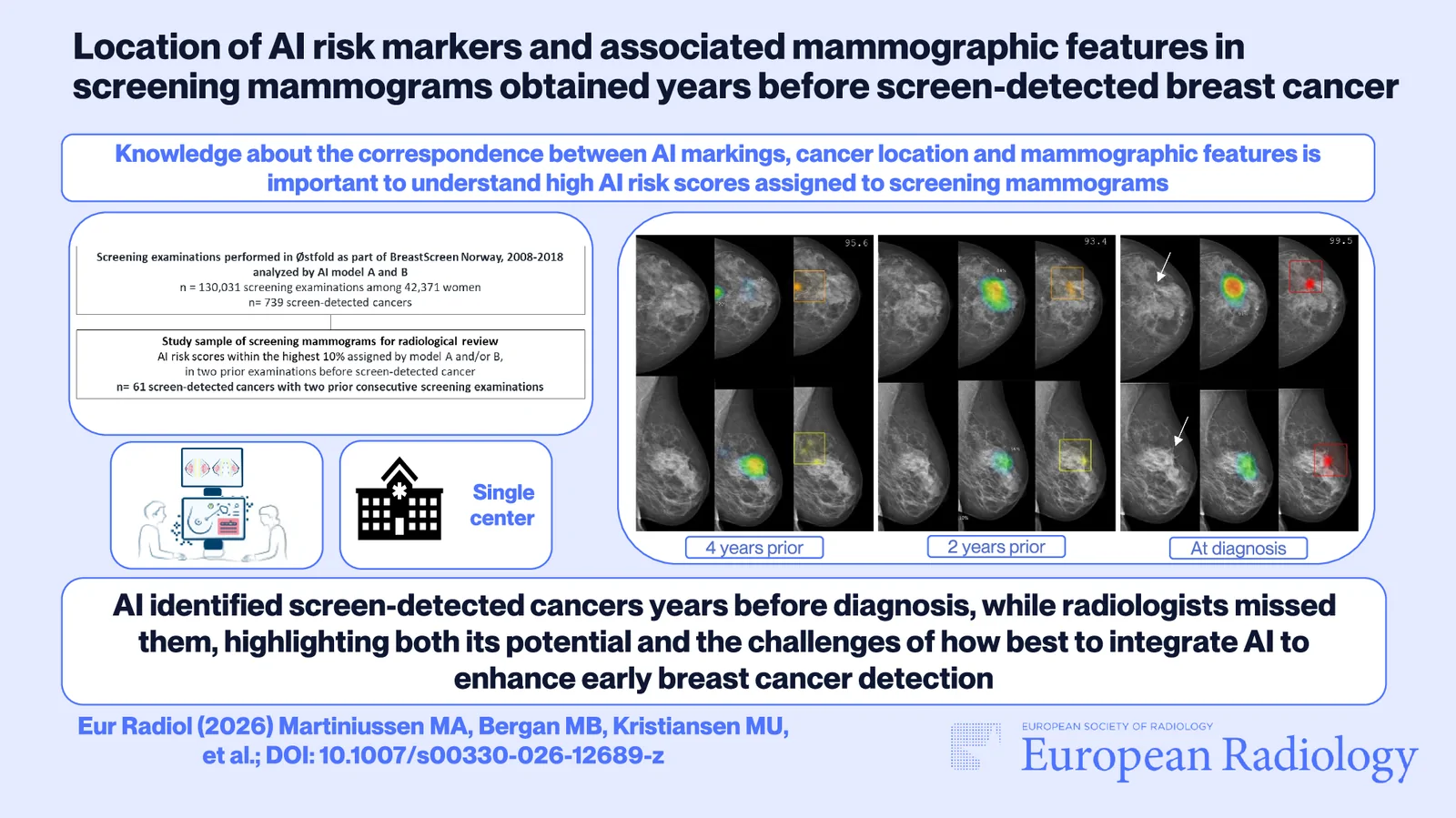

## Introduction

Breast cancer is the most frequently diagnosed cancer and the leading cause of cancer-related death among females globally, with 2.3 million new cases and nearly 666,000 deaths in 2022 [1]. Mammographic screening enables early detection, reducing the incidence of advanced disease, treatment burden [2], and breast cancer-specific mortality [3, 4]. Nevertheless, accurate detection on mammograms remains challenging, and review studies have demonstrated that 20–30% of screen-detected and interval cancers had visible signs on prior mammograms [5, 6]. Moreover, mammographic screening is associated with potential harms, including overdiagnosis and false positive results, which may result in unnecessary anxiety and interventions [7, 8]. Enhancing both sensitivity and specificity, together with improved workflow strategies, is therefore essential to optimize the benefits of screening programs.

Artificial intelligence (AI) models, particularly those based on deep learning, have demonstrated potential to enhance early breast cancer detection, assigning high AI risk scores on screening mammograms preceding interval cancers and next-round screen-detected cancers [9–14]. These models typically generate a malignancy risk score at examination and breast level and mark regions of interest. However, a high AI risk score does not necessarily imply correlation between the AI markings and cancer location [15–18]. A Swedish study reported AI markings corresponding to the later diagnosed cancer in half of the screening examinations preceding interval cancer with score 10 (1–10 scale) and retrospectively classified by radiologists as having minimal signs of malignancy [17]. Despite growing interest in AI-based mammographic analysis, limited knowledge exists regarding how AI models respond to specific mammographic features [19–22]. Feature characterization remains central in radiologic interpretation. The Breast Imaging and Data System (BI-RADS) provides a standardized lexicon and assessment framework that supports consistent evaluation and communication of mammographic findings and corresponding malignancy risk [23], and is widely adopted by breast radiologists.

Two retrospective studies have concluded that AI models may identify breast cancer 2 and 4 years prior to diagnosis, but neither the spatial correspondence of AI markings and tumor locations nor the specific features contributing to the AI risk score were examined [11, 12].

To address some of the knowledge gaps related to high AI risk scores, we conducted a review of screen-detected cancers and screening mammograms assigned AI risk scores within the highest 10% in the screening rounds 4 and 2 years prior to diagnosis. We examined the locations of AI markings, mammographic features, and classified whether there were signs of malignancy prior to diagnosis.

## Materials and methods

The study was approved by the Regional Committees for Medical and Health Research Ethics (#13294, #11022) and conducted in compliance with GDPR Articles 6 (1) (e) and 9 (2) (j). Data disclosure was legally authorized under the Personal Health Filing system Act Section 19a-h [24] and the Cancer Registry Regulation Section 3–1 [25].

### BreastScreen Norway

BreastScreen Norway, managed by the Cancer Registry, Norwegian Institute of Public Health (NIPH), invites approximately 680,000 women aged 50–69 years to two-view (craniocaudal and mediolateral oblique views) digital mammography biennially. The screening mammograms are independently interpreted by two radiologists, who assign a score from 1 to 5, where 1 indicates normal findings and 5 indicates high suspicion of malignancy. Examinations assigned a score ≥ 2 by one or both radiologists are reviewed in a consensus/arbitration meeting to determine whether recall for further assessment is warranted. During the period 2017–2021, the attendance was 76%, recall 3.3%, screen-detected cancer 6.2 per 1000, and interval cancer 1.8 per 1000 screening examinations in the program [26].

### The AI models

We used two AI models trained for breast cancer detection in the study. Model A was Lunit INSIGHT MMG version 1.1.7.2, a commercially available model (Conformité Européenne-marked (CE-marked) and cleared by the Food and Drug Administration [FDA]). The model generated a continuous breast level malignancy risk score ranging from 0 to 100%, with higher scores indicating a greater likelihood of cancer. Mammograms with scores ≥ 10% included one or more heatmap markings, each associated with a local risk score [27].

AI model B was an in-house model, developed by the Norwegian Computing Center (NCC) in collaboration with the Cancer Registry, NIPH. The model is described elsewhere [28]. Importantly, mammograms from Østfold county were not included in the training or cross-validation of this model. Model B produced continuous risk scores from 0 to 100 and highlighted square regions of interest in each view, color-coded from green to red to indicate increasing risk of breast cancer. All regions of interest except those labeled green were accompanied by one or more markings. The overall performance of both AI models has been reported for a population largely overlapping with the present study cohort [18].

### Study sample

This retrospective study included data from 130,031 screening examinations among 42,371 women, screened at Østfold Hospital as part of BreastScreen Norway in the period from January 2, 2008, to December 19, 2018. All mammograms were pseudonymized and analyzed using AI models A and B. After exclusions, 129,385 examinations, including 749 screen-detected cancer were eligible for analysis. Mean age at diagnosis was 62.0 years (SD = 4.5).

**Fig. 1:**
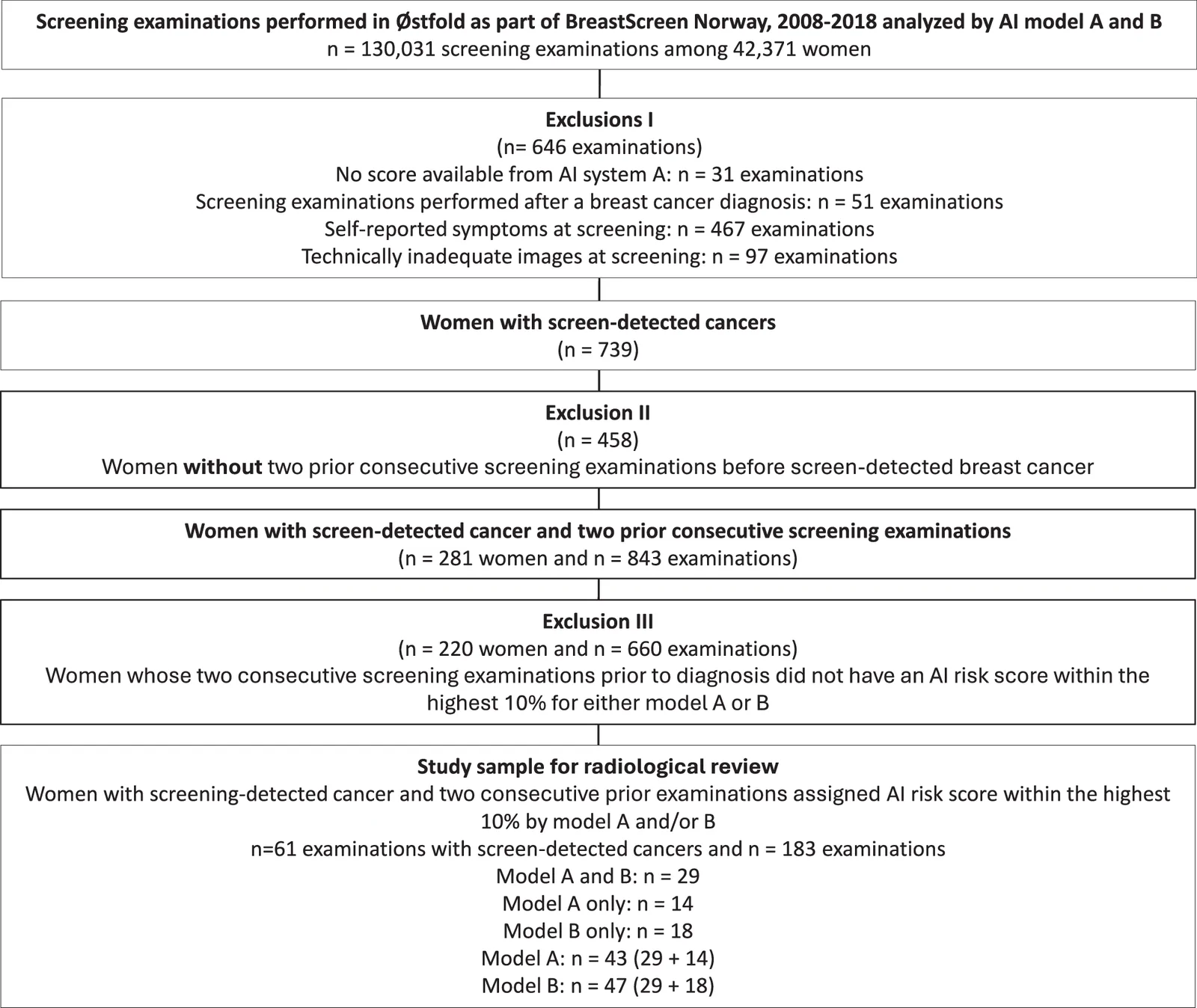
Flowchart of the study sample

The study sample included women with screen-detected cancer and two consecutive screening examinations prior to diagnosis, where at least one of the two AI models had assigned a risk score within the highest 10% at both prior examinations, forming sets of three consecutive screening examinations: conducted 4 and 2 years prior to, and at diagnosis (Fig. 1).

### Variables of interest

Screen-detected cancer was defined as breast cancer diagnosed after recall assessment due to mammographic findings and included histologically verified ductal carcinoma in situ (DCIS) and invasive cancer. An examination was considered selected when assigned a high AI risk score, defined as a risk score within the highest 10%, among the 129,385 screening examinations in the study population, assigned by AI model A and/or B. The AI models were considered stand-alone readers of retrospective data. The mammogram at diagnosis was defined as the screening mammogram leading to a recall and the screen-detected cancer diagnosis.

### Radiological review

Two breast radiologists (M.U.K. and M.A.M., 21 and 8 years of screen reading experience, respectively) conducted a consensus-based informed review of three consecutive two-view screening mammograms from women with screen-detected cancer and examinations flagged as high risk by the AI systems 4 and 2 years prior to diagnosis.

We recorded whether the markings corresponded to the cancer location in one or both views at all three time points. An AI marking was considered corresponding if it was within the cancer boundary and the true cancer location was verified using diagnostic mammograms and complementary imaging modalities, including tomosynthesis, ultrasound, and MRI, if needed.

Mammographic features at the cancer location were classified according to a modified BI-RADS classification: mass, spiculated mass, asymmetry, distortion, calcification alone, density with calcification and no features [23]. For examinations 4 and 2 years prior to diagnosis, findings at the cancer location were classified into true negative (no findings at the cancer location), minimal sign non-specific (non-specific findings at the cancer location with a recall not considered probable), minimal sign significant (subtle findings not necessarily regarded as warranting a recall), and false negative (obvious findings), according to European guidelines [29].

### Statistical analysis

Stratified by AI model, we reported frequencies and percentages of mammographic views with AI markings corresponding to the cancer location and mammographic features on three consecutive screening mammograms, histopathological tumor characteristics of the screen-detected cancers, and classifications of signs of malignancy on prior screening mammograms. A Sankey diagram illustrated changes in mammographic features across the screening examinations. All analyses were performed using Stata (StataCorp, 2025, Stata Statistical Software: Release 19).

## Results

Among the 281 women with screen-detected cancer and two prior consecutive screening examinations, AI model A and B classified, respectively, 15% (43/281) and 17% (47/281) as high risk (within the highest 10%), 4 and 2 years prior to a screen-detected cancer diagnosis. Twenty-nine cases were selected by both AI models, while 14 cases were selected by model A only and 18 by model B only, a total of 61 screen-detected cancers/sets of three consecutive screening examinations (Fig. 1).

AI markings corresponding to the cancer location in both views 4 years prior to diagnosis were observed in 42% (18/43) for model A and 34% (16/47) for model B (Table 1). Two years prior to diagnosis, the corresponding percentages were 61% (26/43) for model A and 49% (23/47) for model B, and at diagnosis, 86% (37/43) and 81% (38/47) for models A and B, respectively.

**Table 1:** Distribution of AI markings corresponding to the cancer location on the mammograms 4 and 2 years prior to, and at diagnosis, by view, for AI models A and B

	4 years prior		2 years prior		At diagnosis	
View with AI markings corresponding to the cancer location	AI model					
	A (*n* = 43)	B (*n* = 47)	A (*n* = 43)	B (*n* = 47)	A (*n* = 43)	B (*n* = 47)
	*n* (%)	*n* (%)	*n* (%)	*n* (%)	*n* (%)	*n* (%)
CC + MLO	18 (42%)	16 (34%)	26 (61%)	23 (49%)	37 (86%)	38 (81%)
Solely CC	1 (2%)	2 (4%)	5 (12%)	5 (11%)	1 (2%)	1 (2%)
Solely MLO	7 (16%)	9 (19%)	4 (9%)	7 (15%)	4 (9%)	5 (11%)
Correct location in at least one view	26 (61%)	27 (57%)	35 (81%)	35 (75%)	42 (98%)	44 (94%)

Percentages were calculated based on the number of screening examinations assigned an AI risk score among the 10% highest at both priors, by model A and/or model B

**Fig. 2:**
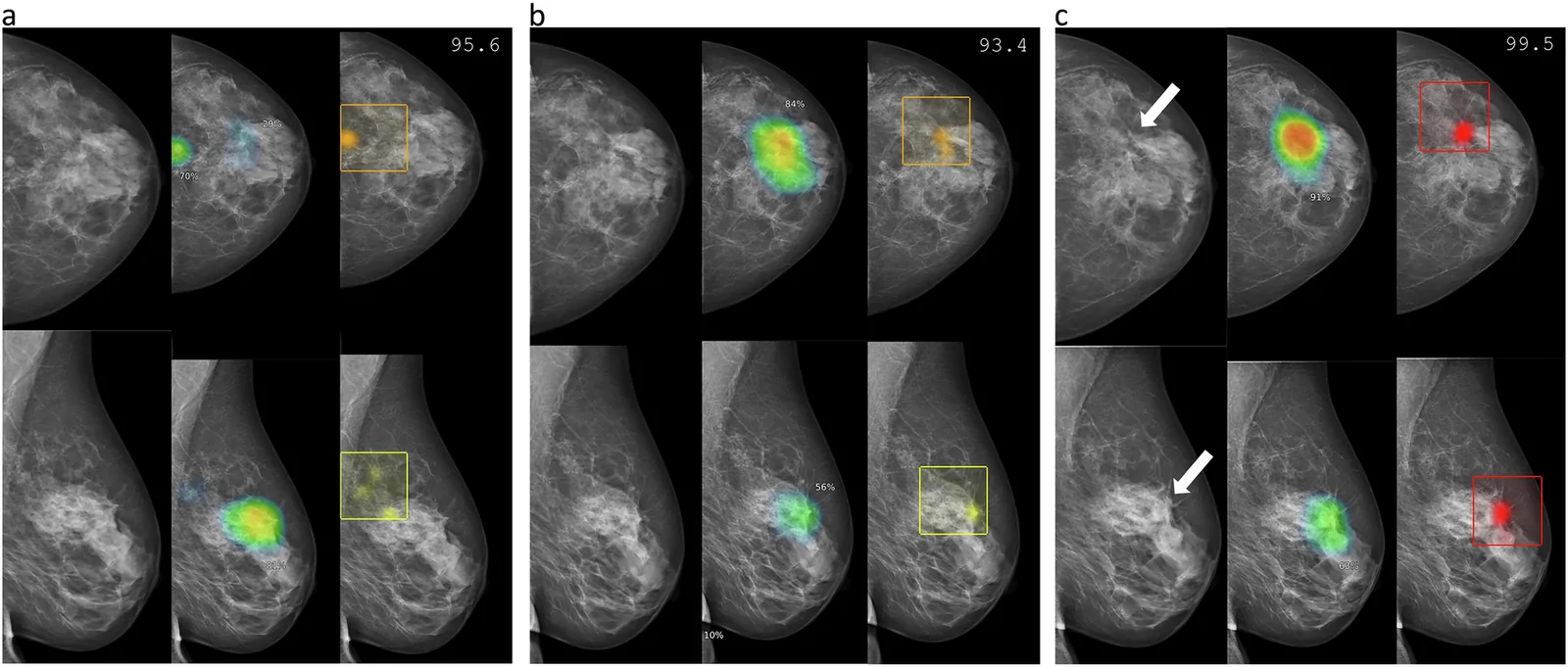
Screening mammograms of the left breast in craniocaudal (CC) and mediolateral oblique (MLO) view as they appear in a clinical setting and with risk scores and markings by AI models A and B. **a** Four years prior to diagnosis, the mammogram was interpreted as negative by the radiologists at screening. Model A marked the cancer location with a high-risk score in the MLO view, whereas in the CC view, the marking at the cancer location was assigned a risk score below the top 10%. The markings by model B did not correspond to the cancer location in any view. **b** Two years prior to diagnosis, the mammogram was interpreted as negative by the radiologists at screening but assigned high-risk scores and markings corresponding to the cancer location in both views by both AI models. **c** At diagnosis, a distortion was identified in the upper lateral quadrant (arrows), prompting a recall by the radiologists. Both AI models assigned high-risk scores, and the markings corresponded to the cancer location in both views. At review, the radiologists classified the mammograms 4 and 2 years prior to diagnosis as true negative. Histopathology: Invasive carcinoma of no special type (NST), grade 3, subtype unknown, lymph node negative, tumor size: 12 mm

Correctly located screen-detected cancers in at least one view were observed in 61% (26/43) for model A, and 57% (27/47) for model B, 4 years before diagnosis. Two years prior to diagnosis, the markings corresponded to the cancer location in 81% (35/43) for model A, and 75% (35/47) for model B, increasing to 98% (42/43) and 94% (44/47), respectively, at diagnosis (Table 1). Figure 2 illustrates a screen-detected cancer with markings by both AI models at all three time points.

**Fig. 3:**
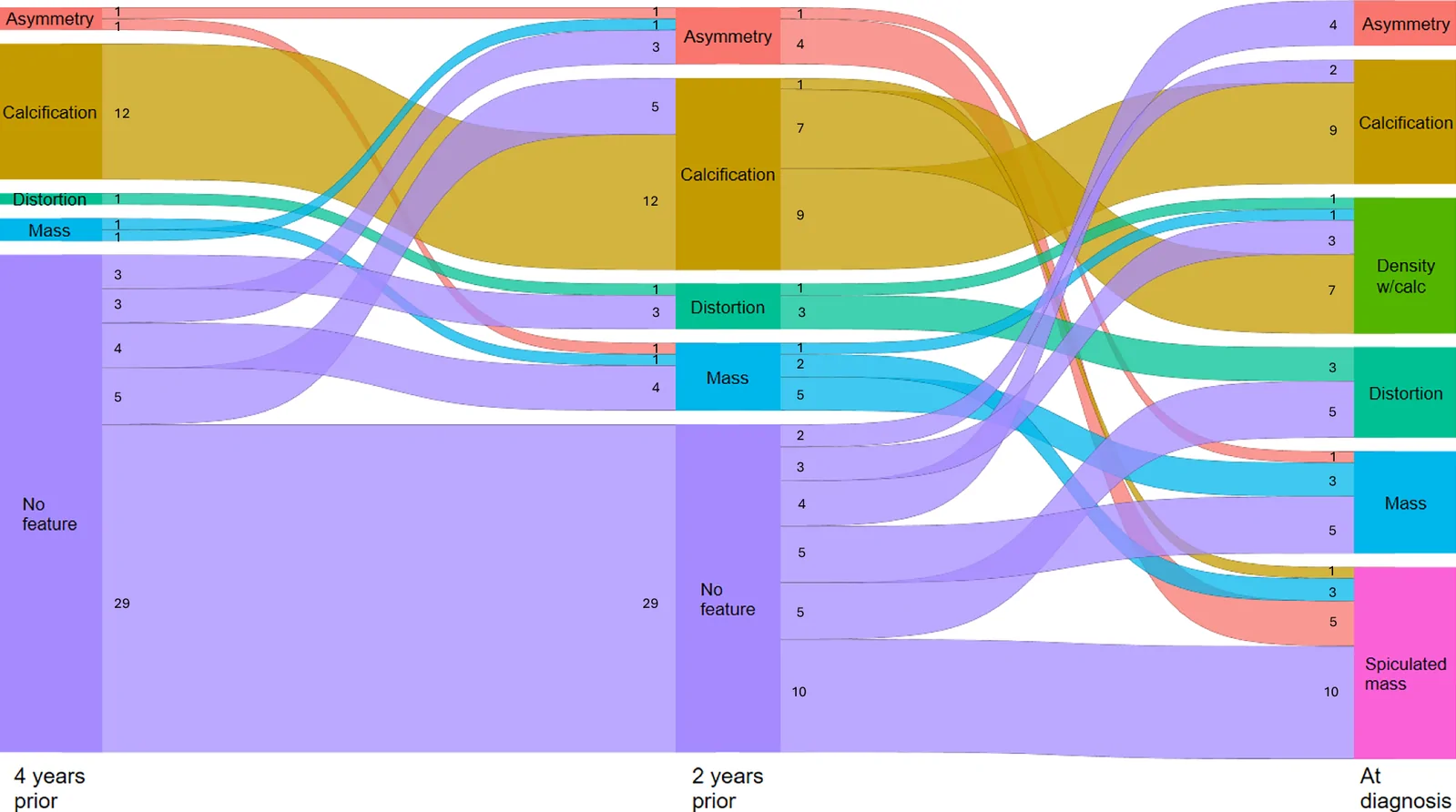
A Sankey diagram illustrates the number of screen-detected cancers and their mammographic features as they appeared on the screening mammograms 4 and 2 years prior to, and at diagnosis. Orange = asymmetry, brown = calcifications (alone), aqua green = distortion, blue = mass, green = density with calcifications, pink = spiculated mass, violet = no feature

Frequencies of mammographic features at the cancer location at all three time points are presented in Supplementary Table 1. Four years before diagnosis, 73% (44/61) of the cases were characterized as having no mammographic feature, while that proportion was reduced to 47% (29/61) 2 years prior, and not reported at diagnosis. Calcifications alone were observed in 19% (12/61) 4 years prior to and in 28% (17/61) 2 years prior to diagnosis, and 18% (11/61) at diagnosis. The most frequent mammographic features at diagnosis were spiculated mass (28%, 17/61) and density with calcifications (20%, 12/61), both absent features 4 and 2 years prior. Among the cases presenting as density with calcification at diagnosis (*n* = 12), seven were classified as calcifications 2 years prior and two cases 4 years prior to diagnosis, while three and nine cases were characterized with no features, 4 and 2 years prior to diagnosis, respectively (Fig. 3).

In all cases where the AI markings did not correspond to the later presenting cancer, the radiologists characterized the cancer location with no features. Among cases with markings corresponding to the cancer location in at least one view, 39% (10/26) were characterized with no feature for model A, and 48% (13/27) for model B, 4 years prior to diagnosis, while the corresponding percentages were 23% (8/35) and 26% (9/35) for model A and B, respectively, 2 years prior to diagnosis (Supplementary Table 2).

Mammographic features of screen-detected cancers stratified by AI model (Table 2), revealed calcifications alone in 29% (4/14) of cases selected by model A only, in no cases selected by model B only, while this feature was present in 24% (7/29) of the cases selected by both models. Spiculated mass was the predominant feature among cancers selected by model B only (9/18), while it was about one-fourth (3/14) of those selected by model A only. Among the 12 cases characterized as density with calcifications, nine (75%) were selected by both models.

**Table 2:** Mammographic features and histopathological tumor characteristics of screen-detected cancers, stratified by AI model (selected by model A only, model B only, and by both models)

Screen-detected cancers	Selected by model A only *n* = 14	Selected by model B only *n* = 18	Selected by models A and B *n* = 29
	*n* (%)	*n* (%)	*n* (%)
Mammographic features			
Mass	3 (21%)	4 (23%)	2 (7%)
Spiculated mass	3 (21%)	8 (45%)	6 (21%)
Asymmetry	1 (7%)	2 (11%)	1 (3%)
Architectural distortion	2 (15%)	2 (11%)	4 (14%)
Calcifications alone	4 (29%)	0 (-)	7 (24%)
Density with calcifications	1 (7%)	2 (10%)	9 (31%)
Histopathological tumor characteristics			
Ductal carcinoma in situ	3 (21%)	1 (6%)	4 (14%)
Invasive cancers	11 (79%)	17 (94%)	25 (86%)
Invasive cancers			
Histological type			
Invasive carcinoma of no special type	10 (91%)	14 (82%)	23 (92%)
Invasive lobular carcinoma	1 (9%)	2 (12%)	1 (4%)
Other invasive carcinomas	0 (-)	1 (6%)	1 (4%)
Tumor diameter (mm), median (IQR)	11 (7–16)	15 (9–25)	12 (10–18)
Histological grade			
Grade 1	2 (18%)	3 (18%)	6 (24%)
Grade 2	7 (64%)	12 (71%)	13 (52%)
Grade 3	2 (18%)	2 (12%)	6 (24%)
Lymph node positive	2 (18%)	6 (35%)	6 (24%)
Immunohistochemical subtypes			
Luminal A-like	9 (82%)	14 (82%)	13 (54%)
Luminal B-like, Her2-	0	1 (6%)	6 (25%)
Luminal B-like, Her2+	1 (9%)	0	3 (13%)
Her2+	1 (9%)	1 (6%)	1 (4%)
Triple negative	0	1 (6%)	1 (4%)
Information not available	0	0	1

Percentages were calculated based on the number of cases selected by model A only, model B only, or by both models

Invasive carcinoma of no special type was the most frequent histological type for all groups; 10 of the 14 cases selected by AI model A only, 14 of the 18 cases selected by model B only, and 24 of the 29 cancers selected by both models. Median tumor diameter was 11 mm for the cancers selected by model A only, 15 mm for those selected by model B only, and 12 mm for those selected by both models. Among the 14 cancers selected by model A only, 2 were lymph node positive, while it was 7 of the 18 cancers selected by model B only and 6 of the 29 selected by both models (Table 2).

**Fig. 4:**
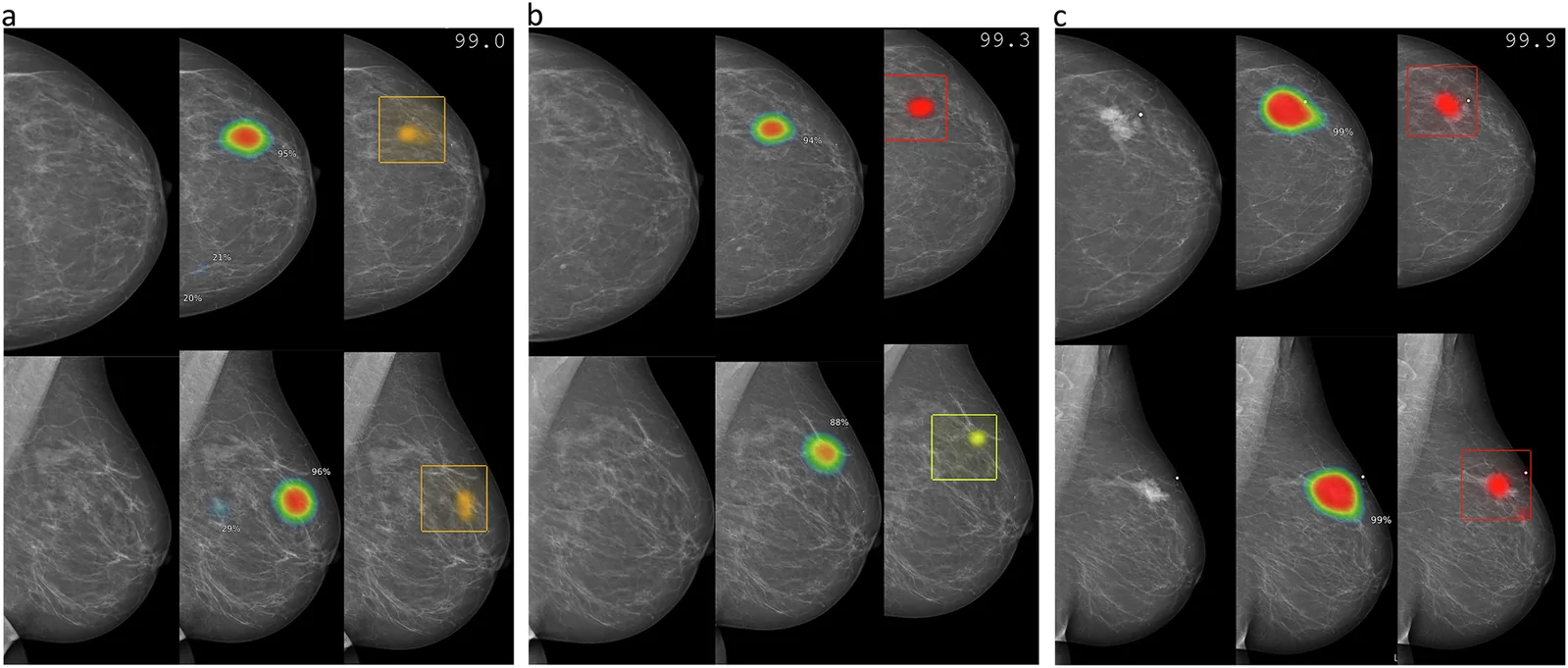
Screening mammograms of the left breast in craniocaudal (CC) and mediolateral oblique (MLO) views as they appear at screening and with risk scores and markings by AI models A and B. **a** Both AI models assigned markings corresponding to the cancer location in both views on mammograms obtained 4 years prior to diagnosis. **b** Markings from both AI models corresponded to the cancer location in both views on mammograms obtained 2 years prior to diagnosis. **c** At diagnosis, a density with calcification was identified in the upper lateral quadrant. AI markings from both models corresponded to the cancer location in both views. At review, the radiologists classified the mammograms 4 and 2 years prior to diagnosis as minimal sign non-specific. Histopathology: Invasive carcinoma of no special type (NST), grade 3, Luminal B-like Her2+, lymph node positive, tumor size 25 mm

**Fig. 5:**
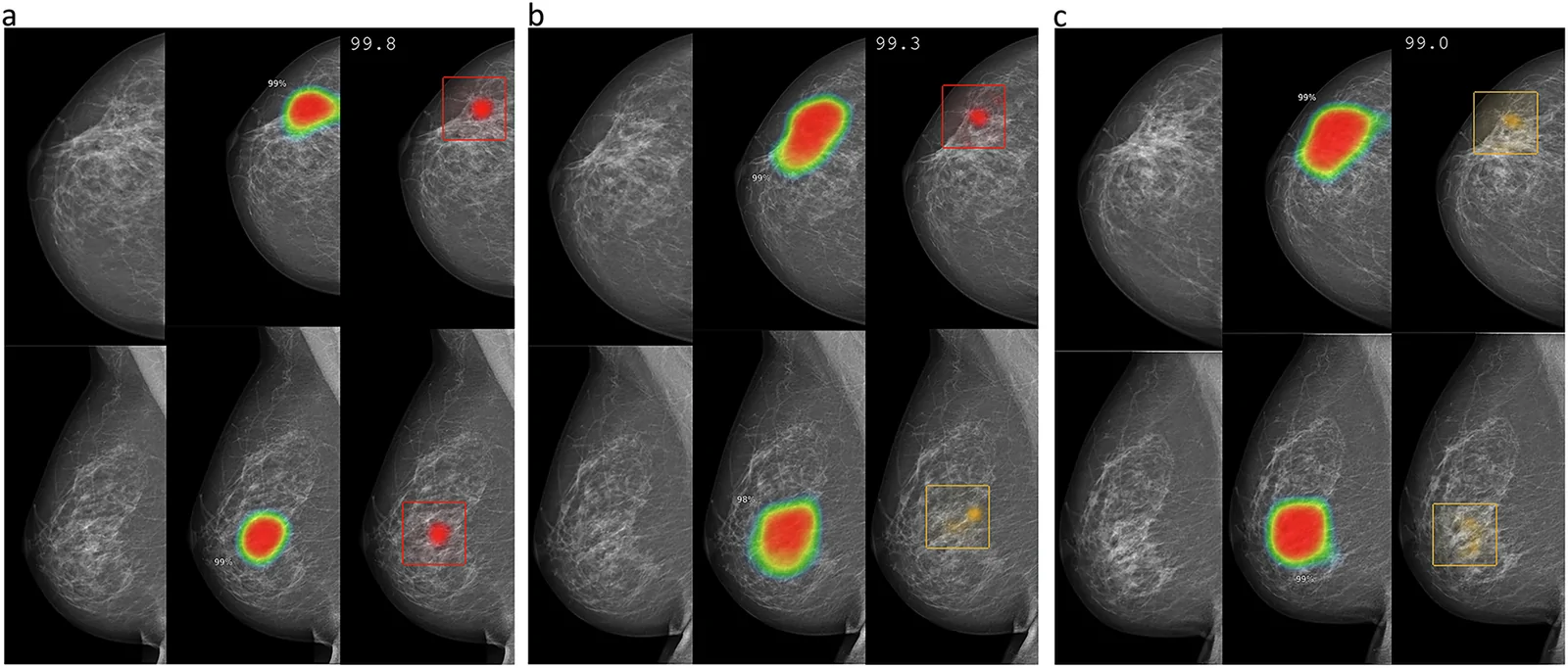
Screening mammograms of the right breast in craniocaudal (CC) and mediolateral oblique (MLO) views as they appear at screening  and with risk scores and markings by AI models A and B. **a** Both AI models marked the cancer location in both views on mammograms obtained 4 years prior to diagnosis. **b** Markings from both AI models corresponded to the cancer location in both views 2 years prior to diagnosis. **c** At diagnosis, calcifications were identified laterally at the level of the mamilla. AI markings from both models corresponded to the cancer location in both views. Radiologists classified the examinations 4 and 2 years prior to diagnosis as false negative. Histopathology: Invasive carcinoma of no special type (NST), grade 3, Luminal B-like Her2-, lymph node positive, tumor size 36 mm

The radiologists classified all screening mammograms with AI markings not corresponding to the cancer locations as true negative, at both prior time points. For the examinations with correctly located AI markings 4 years before diagnosis, 89% (23/26) selected by model A and 89% (24/27) selected by model B, were classified as true negative (Fig. 2) or as minimal sign non-specific (Fig. 4) by the radiologists. Two years prior to diagnosis, the corresponding proportions were 89% (31/35) for AI model A and 83% (29/35) for model B (Table 3). Figure 5 demonstrates a case classified as false negative 4 and 2 years prior to diagnosis.

**Table 3:** Classification of signs of malignancy at the location corresponding to the later diagnosed cancer, 4 years and 2 years prior to the screen-detected cancer diagnosis, stratified by AI markings not corresponding and corresponding to the cancer location in at least one view by AI models A and B

Classification	AI model A		AI model B	
	AI markings not corresponding or corresponding to the cancer location			
	Not corresponding	Corresponding	Not corresponding	Corresponding
	*N* (%)	*N* (%)	*N* (%)	*N* (%)
4 years prior to diagnosis	*n* = 17	*n* = 26	*n* = 20	*n* = 27
True negative	17 (100%)	13 (50%)	20 (100%)	15 (56%)
Minimal sign non-specific	0 (-)	10 (39%)	0 (-)	9 (33%)
Minimal sign significant	0 (-)	2 (8%)	0 (-)	2 (7%)
False negative	0 (-)	1 (3%)	0 (-)	1 (4%)
2 years prior to diagnosis	*n* = 8	*n* = 35	*n* = 12	*n* = 35
True negative	8 (100%)	9 (26%)	12 (100%)	12 (34%)
Minimal sign non-specific	0 (-)	22 (63%)	0 (-)	17 (49%)
Minimal sign significant	0 (-)	3 (8%)	0 (-)	4 (11%)
False negative	0 (-)	1 (3%)	0 (-)	2 (6%)

The classifications were determined in a radiological consensus review

We stratified histopathological characteristics by model and radiologists’ classifications, among examinations in which the AI markings corresponded to the cancer location (Supplementary Table 3).

## Discussion

In this retrospective study of screen-detected cancers assigned high risk scores (top 10%) by two AI models at two prior consecutive examinations, the models correctly located the cancers in 61% and 57% of the cases, respectively, 4 years prior to diagnosis. However, radiologists classified 89% of these as true negative or minimal sign non-specific. This suggests that the AI models may have the potential to identify subtle pathological patterns on mammograms, not possible to capture by the radiologists.

AI markings corresponded with the cancer location in at least one view in 61% and 57% and in both views in 42% and 34% of the cases for models A and B, respectively, 4 years prior to diagnosis. Radiologists may interpret an examination as positive based on a suspicious finding in a single view, as they are well aware that some breast cancers are visible in only one view. When AI markings correspond with suspicious mammographic features, correspondence across views is unlikely to affect recall. However, for very subtle signs of cancer, typically interpreted as negative by radiologists, high risk scores with corresponding markings in two views may be considered more suspicious than findings marked in only one view, and could influence radiologists’ interpretation and recall decision.

Both AI models assigned high risk scores and correctly localized cancers years before diagnosis in a similar number of cases where radiologists reported no signs of malignancy. This discrepancy represents a challenge regarding how best to leverage AI’s potential in early detection of breast cancers and underscores the need to optimize how AI outputs are incorporated into screening workflows. At a threshold where the top 10% of AI risk scores are considered AI-positive, most examinations, about 93%, will not represent cancer. Additional imaging modalities, such as contrast-enhanced mammography or MRI, could increase early detection due to higher sensitivity. However, offering supplemental imaging solely based on a high AI risk score would dramatically increase recall rates, impose psychological burden from false positive examinations, and strain screening resources. Thus, AI may be valuable not only for cancer detection but also for risk stratification in personalized screening [30]. Supporting this, Gjesvik et al reported higher mean AI scores in examinations 2 and 4 years prior to a screen-detected cancer compared to non-cancer cases [12]. Future AI-based risk models incorporating mammographic density, texture features, and established risk factors such as family history and lifestyle may enable more tailored screening strategies. High-risk women could undergo more frequent examinations and benefit from additional imaging, while low-risk women could be screened less often. Improved AI differentiation between benign and malignant features may also lower the threshold for AI-positive exams, reducing the number flagged as suspicious. Mammographic features evolved over time, with spiculated masses and density with calcifications emerging as the most frequent findings at diagnosis, despite being absent 4 and 2 years prior. Among cancers presenting as density with calcification at diagnosis, 59% appeared as calcifications alone 2 years prior. Interpreting calcifications is challenging, as most are benign, although clusters on screening mammograms have been associated with an increased breast cancer risk, compared to mammograms without [31]. Moshina et al further reported an increased risk of breast cancer death among women whose cancers presented as densities with calcifications compared with spiculated masses [32], underscoring the importance of early detection. AI models have been shown to assign high risk scores to mammograms containing calcifications, including both malignant and small benign clusters [19, 33]. This highlights the need for improved AI-based differentiation and thorough radiological knowledge of benign versus malignant calcification patterns.

There was no complete overlap between examinations selected by the different AI models, and stratifying mammographic and histopathological features by AI model revealed differences in the cases each selected. Using two AI models increased the number of cases identified prior to diagnosis, a finding supported by a Korean study using two commercially available AI models [34]. However, in a clinical setting, this would also increase AI-generated false positive examinations, and evaluation of the benefits, drawbacks, and economic implications of such an approach is needed. Comparative evaluation of model performance may enhance understanding of their respective strengths and limitations, potentially improving radiological trust and quality assurance.

The use of data from a regular screening setting is a strength of this study. Limitations included the retrospective single-center design, where the classification in the review might have been biased due to the use of two internal radiologists. The classification categories used to assess mammographic signs of malignancy in examinations prior to diagnosis may be subject to variable interpretation, potentially reducing the generalizability of study findings. The study sample included cases with high AI risk scores at two screening rounds preceding screen-detected cancer. This approach may have introduced selection bias, as screen-detected cancers showing early malignant signs years before diagnosis may not be representative of all screen-detected cancers. Including examinations with high scores only 2 years prior to diagnosis, or those preceding interval cancers, would have increased the number of cases and potentially included cancers with different characteristics, possibly yielding different results. Such an approach might better reflect a real-world setting. It would have been clinically relevant to assess whether cases showing mammographic signs of malignancy before diagnosis differed histopathologically from those without. However, the number of these cases was too small to draw conclusions.

In conclusion, this study indicates potential for AI-assisted mammographic screening to enhance early breast cancer detection, as two separate AI systems flagged prior mammograms as high risk 2 and 4 years before diagnosis. Because AI-marked regions often lack suspicious findings visible to radiologists, the true clinical impact of AI-assisted screening remains uncertain. Further retrospective and prospective studies are therefore warranted. These findings also suggest a potential role for AI in risk stratification and in the development of more personalized breast cancer screening strategies.

## Supplementary information

Supplementary information

## Abbreviation

AI: Artificial intelligence
