# Supplementary material for: Location of AI risk markers and associated mammographic features in screening mammograms obtained years before screen-detected breast cancer
Source: Eur Radiol. 2026 Jun 18;36(11):8380–90. doi: 10.1007/s00330-026-12689-z (PMC13661703; doi:10.1007/s00330-026-12689-z)
Supplement: Supplementary file 1 [file 330_2026_12689_MOESM1_ESM.pdf]

Location of AI risk markers and associated mammographic features in screening mammograms obtained years before screen-detected breast cancer

ELECTRONIC SUPPLEMENTARY MATERIAL

Supplementary table 1. Mammographic features at the location corresponding to the screen-detected cancers, four and two years prior to, and at diagnosis in examinations with high AI risk score (top 10%) assigned by AI model A, model B or both, at both prior examinations.

Mammographic features	4 years prior (N=61)	2 years prior (N=61)	At diagnosis (N=61)
	n (%)	n (%)	n (%)
Mass	2 (3%)	6 (10%)	9 (15%)
Spiculated mass	0 (-)	0 (-)	17 (28%)
Asymmetry	2 (3%)	5 (8%)	4 (6%)
Architectural distortion	1 (2%)	4 (7%)	8 (13%)
Calcifications alone	12 (19%)	17 (28%)	11 (18%)
Density with calcifications	0 (-)	0 (-)	12 (20%)
No features	44 (73%)	29 (47%)	0 (-)

Supplementary table 2. Mammographic features associated with the screen-detected cancer location on screening mammograms two- and four years prior to diagnosis, stratified by AI markings not corresponding and corresponding with cancer location in at least one view by model A and B. The characterizations were determined in a consensus radiological review.

	Model A<br>Mammographic features at the cancer location		Model B<br>Mammographic features at the cancer location	
	AI markings not corresponding or corresponding to the cancer location			
	Not corresponding	Corresponding	Not corresponding	Corresponding
**4 years prior to diagnosis**	N=17	N=26	N=20	N=27
	n (%)	n (%)	n (%)	n (%)
Mass	0 (-)	2 (8%)	0 (-)	0 (-)
Spiculated mass	0 (-)	0 (-)	0 (-)	0 (-)
Asymmetry	0 (-)	1 (4%)	0 (-)	2 (7%)
Architectual distortion	0 (-)	1 (4%)	0 (-)	1 (4%)
Calcifications alone	0 (-)	12 (46%)	0 (-)	11 (41%)
Density with calcifications	0 (-)	0 (-)	0 (-)	0 (-)
No features	17 (100%)	10 (39%)	20 (100%)	13 (48%)
**2 years prior to diagnosis**	N=8	N=35	N=12	N=35
Mass	0 (-)	2 (6%)	0 (-)	5 (13%)
Spiculated mass	0 (-)	0 (-)	0 (-)	0 (-)
Asymmetry	0 (-)	5 (14%)	0 (-)	3 (9%)
Architectual distortion	0 (-)	3 (9%)	0 (-)	4 (11%)
Calcifications alone	0 (-)	17 (49%)	0 (-)	14 (40%)
Density with calcifications	0 (-)	0 (-)	0 (-)	0 (-)
No features	8 (100%)	8 (23%)	12 (100%)	9 (26%)

Supplementary table 3. Histopathological characteristics of screen-detected cancers, stratified by AI model and radiologists' classification of early signs of malignancy. The table includes cases within the 10% highest of AI risk scores four and two years prior to diagnosis, limited to cases with corresponding AI markings with the cancer location four and/or two years prior to diagnosis.

	Model A		Model B	
Histopathological tumor characteristics	Radiologists' classification			
	True negative/ minimal sign non specific	Minimal sign significant/ false negative	True negative/ minimal sign non specific	Minimal sign significant/ false negative
4 years prior to diagnosis	n=23	n=3	n=24	n=3
Ductal carcinoma in situ	3 (13%)	0 (-)	3 (13%)	0 (-)
Invasive cancers	20 (87%)	3 (100%)	21 (88%)	3 (100%)
Invasive Cancers				
Histological type				
Invasive carcinoma of no special type	20 (100%)	3 (100%)	19 (90%)	3 (100%)
Invasive lobular carcinoma	0 (-)	0 (-)	1 (5%)	0 (0%)
Other invasive carcinomas	0 (-)	0 (-)	1 (5%)	0 (0%)
Tumor diameter (mm), median (IQR)	12 (8-15)	9 (8-65)	12 (9-16)	9 (8-65)
Histological grade				
Grade 1	5 (25%)	2 (67%)	5 (24%)	2 (67%)
Grade 2	10 (50%)	0 (-)	12 (57%)	0 (-)
Grade 3	5 (25%)	1 (33%)	4 (19%)	1 (33%)
Lymph node positive	4 (20%)	1 (33%)	6 (29%)	1 (33%)
Immunohistochemical subtypes				
Luminal A-like	10 (53%)	2 (67%)	12 (57%)	2 (67%)
Luminal B-like, Her2-	4 (21%)	1 (33%)	4 (19%)	1 (33%)
Luminal B-like, Her2+	3 (16%)	0 (-)	2 (10%)	0 (-)
Her2+	2 (11%)	0 (-)	2 (10%)	0 (-)
Tripple negative	0 (-)	0 (-)	1 (5%)	0 (-)
Information not available	1	0	0	0
2 years prior to diagnosis	n=31	n=4	n=29	n=6
Ductal carcinoma in situ	2 (6%)	2 (50%)	2 (7%)	2 (33%)
Invasive cancers	29 (94%)	2 (50%)	27 (93%)	4 (67%)
Invasive Cancers				
Histological type				
Invasive carcinoma of no special type	28 (97%)	2 (100%)	24 (89%)	3 (75%)
Invasive lobular carcinoma	1 (3%)	0 (0%)	3 (11%)	0 (-)
Other invasive carcinomas	0 (-)	0 (-)	0 (-)	1 (25%)
Tumor diameter (mm), median (IQR)	12 (9-15)	37 (9-65)	12 (9-18)	10 (8-38)
Histological grade				
Grade 1	7 (24%)	1 (50%)	6 (22%)	2 (50%)
Grade 2	17 (59%)	0 (0%)	16 (59%)	1 (25%)
Grade 3	5 (17%)	1 (50%)	5 (19%)	1 (25%)
Lymph node positive	6 (21%)	1 (50%)	7 (26%)	2 (50%)
Immunohistochemical subtypes				
Luminal A-like	18 (64%)	1 (50%)	17 (65%)	3 (75%)
Luminal B-like, Her2-	4 (14%)	1 (50%)	4 (15%)	1 (25%)
Luminal B-like, Her2+	4 (14%)	0 (0%)	3 (12%)	0 (-)
Her2+	2 (7%)	0 (0%)	2 (8%)	0 (-)
Tripple negative	0 (-)	0 (-)	0 (-)	0 (-)
Information not available	1	0	1	0
